# Analysis of prognostic risk factors and risk management measures for patients with ischemic stroke and bloodstream infection based on machine learning

**DOI:** 10.3389/fcimb.2025.1715309

**Published:** 2026-01-27

**Authors:** Xiaojun Li, Zhihui Liang, Aiyu Zhang, Shaoqin Lai, Yan Duan, Chuangchuang Mei, Xiaojing Hong, Donghao Cai, Taoyuan Huang

**Affiliations:** 1 Administration Department of Nosocomial Infection, Guangdong Provincial Second Hospital of Traditional Chinese Medicine, Guangzhou, Guangdong, China; 2 Guangdong Provincial Key Laboratory of Research and Development in Traditional Chinese Medicine, Guangzhou, Guangdong, China; 3 Clinical Laboratory, Guangzhou Xinhai Hospital, Guangzhou, Guangdong, China; 4 Administration Department of Nosocomial Infection, The People's Hospital of Baiyun District Guangzhou, Guangzhou, Guangdong, China; 5 Clinical Laboratory, Guangzhou Twelfth People’s Hospital, Guangzhou, Guangdong, China; 6 Clinical Laboratory, Guangdong Provincial Second Hospital of Traditional Chinese Medicine, Guangzhou, Guangdong, China; 7 Department of Hospital Office, Guangdong Provincial Second Hospital of Traditional Chinese Medicine, Guangzhou, Guangdong, China

**Keywords:** machine learning, LASSO regression, ischaemic stroke, bloodstream infection, prognostic risk factors, risk management

## Abstract

**Objective:**

Through the machine learning Least absolute shrinkage and selection operator (LASSO) algorithm, the system screened core prognostic risk factors for patients with ischaemic stroke and bloodstream infection, constructed and validated a predictive model, provides a basis for formulating precise risk management strategies in clinical practice.

**Methods:**

Clinical data from patients with bloodstream infections secondary to ischaemic stroke were retrospectively included. The dataset was randomly allocated into training and validation sets at a 7:3 ratio. Within the training set, Model 1 was constructed using traditional univariate and multivariate Cox regression methods, while Model 2 employed the machine learning LASSO regression algorithm. Models were compared using metrics including R-squared, C-index. The superior model was selected for validation on both training and validation sets using Area Under the Curve (AUC) of the receiver operating characteristic curve, calibration curves, and Decision-Making Curves (DCA).

**Results:**

Model 2 was adopted as the final model, with a nomogram generated for the training set. As demonstrated by the nomogram, an increase in the total score was observed in patients with concomitant pneumonia, heart failure (HF), or coronary atherosclerotic heart disease (CHD), in cases where mechanical ventilation (MV) was utilised, and in instances of elevated alanine aminotransferase (ALT) and C-reactive protein-lymphocyte ratio (CLR) values, and reduced albumin levels. In the training set, the AUC values for predicting 7-day, 14-day, and 28-day survival rates were 0.875, 0.886, and 0.861, respectively. The AUC value for the 28-day prognosis on the internal test set was 0.844, while that on the external validation dataset was 0.860. The model demonstrated high concordance between predicted and actual probabilities across three distinct cohorts. The clinical decision curve indicates that the model provides good net benefit within the 5%-25% range at all three time points (7,14,and 28 days) across the three datasets.

**Conclusion:**

Comorbidities such as pneumonia and hypoalbuminaemia constitute prognostic risk factors affecting patients with ischaemic stroke and bloodstream infections. LASSO regression enables precise identification of these risk factors, yielding a prognostic prediction model with outstanding predictive efficacy and clinical utility. Clinicians may utilise model variables to implement targeted risk management strategies.

## 1 Introduction

Ischemic stroke constitutes the predominant form of stroke on a global scale, having become a leading cause of mortality and disability. In 2021, the global incidence of new cases of ischemic stroke was 7.6 million per annum (GBD 2021 Stroke Risk Factor Collaborators, 2024). Furthermore, the number of individuals who had experienced an ischemic stroke was more than 77 million. Accelerating population ageing and rising prevalence of underlying conditions such as hypertension and diabetes are key factors in the increasing incidence of ischaemic stroke, which is now affecting a broader demographic, with this figure increasing annually. It is evident that this places a substantial burden upon healthcare systems and affected families (Feigin et al., 2025). The World Stroke Organisation has predicted that in the absence of an expansion in global stroke interventions, the burden of stroke will continue to increase, thereby a severe threat to the sustainability of global healthcare systems. Implementing targeted risk management measures, such as multidisciplinary rehabilitation services and stroke care services, has been demonstrated to effect a prompt improvement in such circumstances (Feigin et al., 2023).

Ischemic strokes are associated with immunosuppression due to cerebral tissue hypoxia and ischaemia, often accompanied by dysphagia, impaired consciousness, and MV. These factors predispose patients to infection, which under certain conditions may progress to bloodstream infection (Simats and Liesz, 2022; Shi et al., 2018). In the event of a bloodstream infection in patients suffering from ischemic strokes, pathogens and their toxins may disseminate throughout the body via the circulatory system, causing sepsis and triggering severe complications such as systemic inflammatory response syndrome and septic shock. This directly exacerbates the patient’s condition, further deteriorates neurological impairment, and significantly increases mortality risk (Joaquim et al., 2024; Bourhy et al., 2022). The combination of ischaemic stroke and bloodstream infection has become a key factor affecting the prognosis of ischaemic stroke patients, demanding urgent attention from clinical healthcare professionals. In recent years, scholars both domestically and internationally have conducted a series of studies on infections associated with ischaemic stroke. However, extant research predominantly remains at the level of identifying risk factors such as ischaemic stroke-associated pneumonia, with scarce reports on bloodstream infections in ischaemic stroke and their prognosis (Awere-Duodu et al., 2024). Moreover, there is paucity of systematic exploration of risk management strategies, which makes it difficult to effectively guide clinicians in formulating individualised infection prevention, control, and treatment plans.

Machine learning algorithms such as LASSO regression employ regularisation penalties to effectively mitigate the impact of multicollinearity whilst identifying key prognostic variables. This approach eliminates redundant variables, enhances model stability and accuracy, and circumvents model errors arising from variable selection bias inherent in traditional regression analysis. Consequently, it is widely applied in screening risk factors and prognostic model variables (Hepp et al., 2016; Ali et al., 2023). Moreover, visual prognostic assessment tools such as nomograms, constructed based on machine learning models, translate complex computational processes into intuitive scoring systems. Clinicians are required to input key patient indicators to rapidly obtain survival probabilities at different time points, significantly enhancing the clinical utility and operability of the model. Therefore, addressing these considerations, this study collected data on bloodstream infections in patients with ischaemic stroke from Guangdong Provincial Second Hospital of Traditional Chinese Medicine over three consecutive years (January 2022 to December 2024). A predictive model was constructed using LASSO regression to investigate the 28-day prognosis of bloodstream infections in ischaemic stroke patients. This construction of the model is intended to provide a tool for clinical intervention in high-risk populations.

## 2 Methods

### 2.1 Patient selection

A retrospective data collection was conducted on patients with bloodstream infections associated with ischaemic stroke admitted to Guangdong Provincial Second Hospital of Traditional Chinese Medicine between January 2022 and December 2024. Inclusion criteria were as follows:

1. The following study will examine hospitalised patients diagnosed with ischaemic stroke between January 2022 and December 2024;
2. Presence of positive blood culture records.
3. Meeting diagnostic criteria for bloodstream infection (Horan et al., 2008): ①Positive blood culture (one or more pathogenic bacteria); ②Presence of at least one of the following symptoms: fever (>38°C), chills, or hypotension; ③Symptoms, signs, and positive laboratory results unrelated to infection at other sites; ④Exclusion of false-positive results due to sample contamination.

Exclusion criteria were as follows: (1) Duplicate cases were excluded. Where the same patient was diagnosed with multiple bloodstream infections caused by the same bacterial strain, only the initial positive blood culture data were utilised;

(2)Cases with incomplete data were excluded. Patients lacking demographic information, laboratory data, or those lost to 28-day follow-up were excluded. Following data collection via the medical records system and laboratory information system, 14 patients exhibited missing key indicators or were lost to follow-up, resulting in an overall missing data rate of 4.1% (14/338). Given that the missing data rate was <5%, a complete case analysis strategy was employed, excluding these 14 cases with incomplete data.

**Figure 1:**
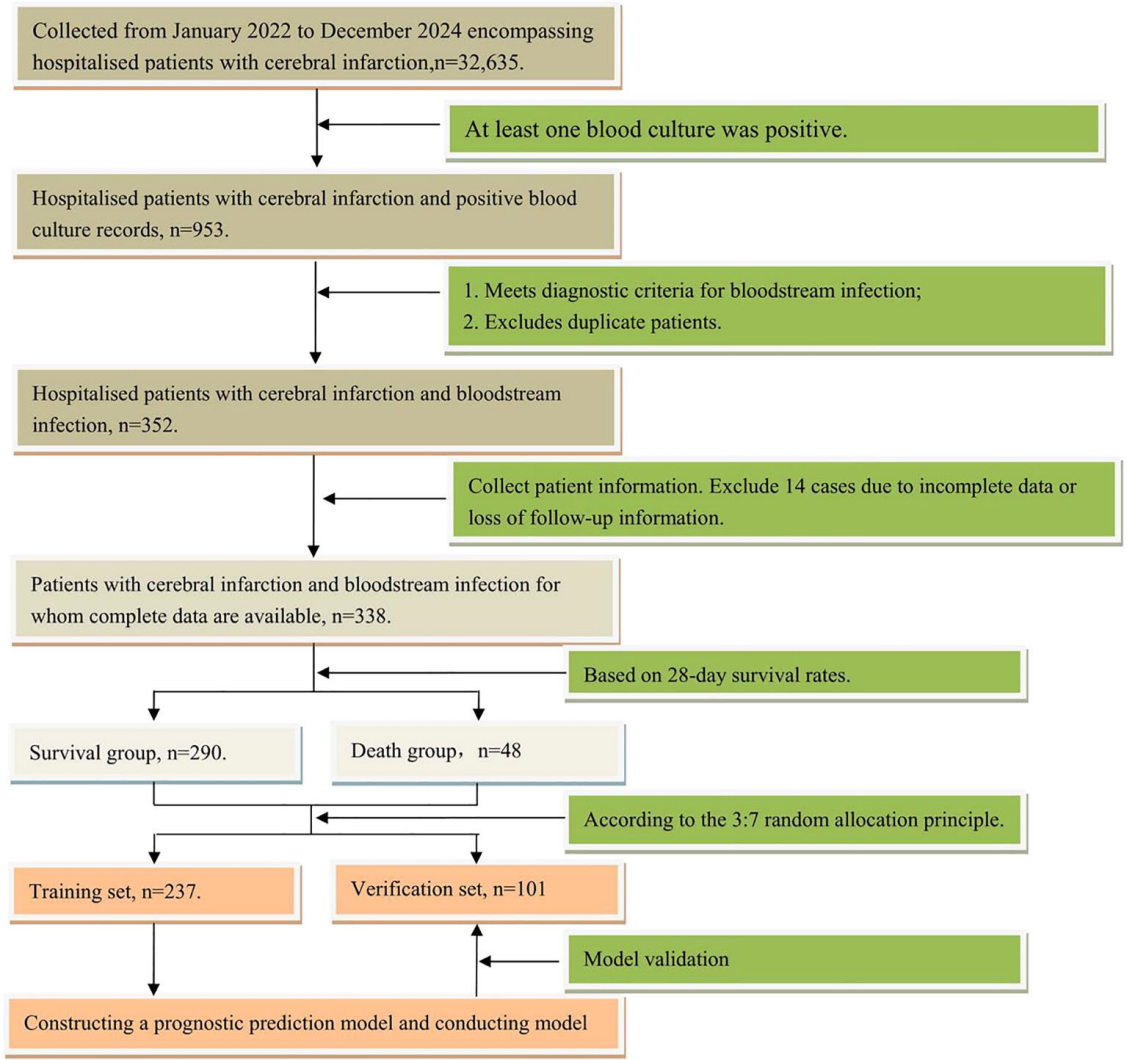
Flowchart of the study on prognosis analysis in patients with ischemic stroke and bloodstream infection.

Following application of inclusion and exclusion criteria, 338 patients were screened. Based on 28-day survival status, these were categorised into a survivor group (n=290) and a mortality group (n=48). The study flowchart is detailed in **Figure 1**. The research received approval from the Ethics Committee of Guangdong Provincial Second Hospital of Traditional Chinese Medicine (Approval No.: Z202404-002-01).

Additionally, 156 cases of bloodstream infections associated with ischaemic stroke from other centres were collected as an external validation dataset, comprising 79 cases from Guangzhou Baiyun District First People’s Hospital, 58 cases from Guangzhou Xinhai Hospital, and 19 cases from Guangzhou Twelfth People’s Hospital.

### 2.2 Data collection

Information was gathered using a self-designed form: this included demographic details such as patient gender and age from the medical records system, alongside underlying conditions including hypertension, pneumonia, HF, and CDH. The hospital infection surveillance system collected data on whether bloodstream infections were mixed infections (defined as blood cultures revealing simultaneous infection by two or more pathogens), whether the infecting organisms were multidrug-resistant organisms (MDR), and whether patients underwent invasive procedures (including urinary catheter (CAU), use of peripherally inserted central venous catheters (PICC), and mechanical ventilation (MV)); From the laboratory information system, collect blood analysis data from the day of infection or within 24 hours, including white blood cell count (WBC), absolute neutrophil count (NEC), absolute lymphocyte count (LYM), absolute monocyte count (MON), haemoglobin (HGB), Platelet count (PLT), C-reactive protein (CRP), Serum albumin (ALB), and other laboratory indicators. Calculate the Systemic Immune Inflammation Index (SII) based on these parameters: Platelet count × Neutrophil count/Lymphocyte count, Neutrophil–lymphocyte ratio (NLR), Platelet–lymphocyte ratio (PLR), Platelet–albumin ratio (PAR), Neutrophil–albumin ratio (NAR). lymphocyte ratio (PLR), platelet-albumin ratio (PAR), neutrophil-albumin ratio (NPAR), lymphocyte-monocyte ratio (LMR), C-reactive protein-albumin ratio (CAR), C-reactive protein-lymphocyte ratio (CLR), C-reactive protein-albumin-lymphocyte index (CALLY index): albumin × lymphocytes ÷ (CRP).

### 2.3 Data analysis

The analysis and modelling of the data was conducted utilising the R software (version 4.3.1). For measures conforming to normal distribution, presented as x ± s, independent samples *t*-tests were employed for intergroup comparisons. Non-normally distributed measures, expressed as median (P25, P75) (M (P25, P75)), underwent intergroup comparisons via Mann-Whitney *U* tests. *P* < 0.05 was considered statistically significant.

All categorical variables were dummy-coded (binary variables were directly included as ‘0=absent, 1=present’; no multi-categorical variables were present), ensuring compliance with regression model input requirements. Preliminary normality testing (Shapiro-Wilk test) of collected continuous variables revealed no extreme skewness in any variable. Furthermore, considering that raw values directly support clinical interpretation and application of scatter plots, all continuous variables were incorporated using their original values without standardisation or distribution transformation. The dataset was randomly allocated into training and validation sets at a 7:3 ratio. The training set was used for model development and evaluation, while the validation set served for model validation. Univariate Cox regression was performed for all variables. Variables with *P* < 0.05 were included in multivariate Cox regression. The Cox model constructed with variables exhibiting *P* < 0.10 in the multivariate Cox regression was termed Model 1. Additionally, LASSO regression was performed on all variables. The penalty term selected via cross-validation was employed to construct Model 2 using the LASSO variable selection method. The Root Mean Squared Error (RMSE), Mean Absolute Error(MAE), and R-squared metrics of both models were compared across the original training set and a dataset subjected to five-fold cross-validation repeated three times. The model demonstrating optimal performance was selected as the final modelling strategy. A nomogram is constructed for the final model, which is then evaluated using AUC, calibration curves, and DCA. The model is subsequently validated on the validation set.

## 3 Results

### 3.1 Patient characteristics

The baseline demographic and clinical characteristics of the training and internal test cohorts are summarised in **Table 1**. Overall, the training cohort consisted of 237 patients, while the internal test cohort included 101 patients. The median age in the training cohort was 76 years (67,85), compared to 79 years (71,87) in the test cohort, with no statistically significant difference between the groups (*p* = 0.163). There was a male predominance in both cohorts, with 60.8% males in the training group and 68.3% in the test group, though this difference was not significant (*p* = 0.188). The distribution of the remaining comorbidities was similar across both groups, and the overall distribution of laboratory blood analysis parameters, ALB,ALT, and other metabolic parameters was comparable between the two groups. The overall comparability of baseline characteristics between the training and testing cohorts supports the validity of using these groups for predictive model development and validation.

**Table 1:** Patient demographics and baseline characteristics.

Characteristic	Cohort			P-value
	Overall N = 338	Training cohort N = 237	Internal test cohort N = 101	
Male, N (%)	213 (63.0%)	144 (60.8%)	69 (68.3%)	0.188
Age, Median (Q1, Q3)	77 (68, 85)	76 (67, 85)	79 (71, 87)	0.163
Combined underlying conditions				
Hypertensive, N (%)	266 (78.7%)	188 (79.3%)	78 (77.2%)	0.666
Pneumonia, N (%)	176 (52.1%)	126 (53.2%)	50 (49.5%)	0.538
HF, N (%)	61 (18.0%)	42 (17.7%)	19 (18.8%)	0.811
CHD, N (%)	91 (26.9%)	62 (26.2%)	29 (28.7%)	0.628
Invasive procedure				
CAU, N (%)	187 (55.3%)	136 (57.4%)	51 (50.5%)	0.244
PICC, N (%)	185 (54.7%)	137 (57.8%)	48 (47.5%)	0.082
MV, N (%)	56 (16.6%)	39 (16.5%)	17 (16.8%)	0.932
Mix infection, N (%)	25 (7.4%)	18 (7.6%)	7 (6.9%)	0.831
MDR, N (%)	168 (49.7%)	125 (52.7%)	43 (42.6%)	0.087
CRP, Median (Q1, Q3)	62.84 (27.1, 125.95)	62.00 (27.64, 126.57)	63.31 (27.04, 101.17)	0.785
WBC, Median (Q1, Q3)	10.62 (6.9, 15.25)	10.98 (7.12, 15.31)	9.90(6.59, 14.51)	0.413
LYM, Median (Q1, Q3)	0.75 (0.45, 1.15)	0.71 (0.44, 1.13)	0.77 (0.46, 1.18)	0.501
MON, Median (Q1, Q3)	0.43 (0.17, 0.73)	0.43 (0.17, 0.73)	0.43 (0.17, 0.65)	0.725
NEC, Median (Q1, Q3)	8.82 (5.53, 13.72)	9.04 (5.57, 13.65)	8.29 (5.51, 13.76)	0.527
HGB, Median (Q1, Q3)	101.5 (80, 120)	100 (79, 120)	103 (82, 120)	0.368
PLT, Median (Q1, Q3)	217 (154.25, 297.5)	224 (156, 296)	205 (144, 301)	0.416
GLU, Median (Q1, Q3)	7.47 (5.97, 10.39)	7.42 (6.05, 10.69)	7.48 (5.54, 9.63)	0.388
CR, Median (Q1, Q3)	79.1 (56.47, 117.33)	79.7 (57, 119)	77.5 (55.8, 115)	0.795
TP, (Mean ± SD)	64.56± 8.46	65.04± 8.43	63.43± 8.45	0.110
ALB, (Mean ± SD)	32.68± 5.61	32.7± 5.63	32.63± 5.59	0.921
ALT. Median (Q1, Q3)	21.1 (11.55, 39.37)	21.1 (11.5, 38)	23.7 (12, 39.4)	0.737
SII, Median (Q1, Q3)	2508.34 (1185.82, 4762.84)	2714.38 (1225.95, 5417.38)	2300.76 (915.4, 3909.24)	0.106
NLR, Median (Q1, Q3)	12.07 (6.09, 22.53)	12.35 (6.49, 24.27)	10.29 (5.56, 19.07)	0.147
PLR, Median (Q1, Q3)	289.88 (185.87, 480.28)	298.46 (178.12, 495.74)	265.33 (198.36, 419.23)	0.310
LMR, Median (Q1, Q3)	1.94 (1.08, 3.97)	1.87 (1.08, 3.6)	1.98 (1.11, 4.51)	0.589
PAR, Median (Q1, Q3)	6.43 (4.83, 9.59)	6.51 (5.06, 9.63)	6.37 (4.53, 8.48)	0.336
NPAR, Median (Q1, Q3)	0.27 (0.16, 0.44)	0.28 (0.17, 0.44)	0.26 (0.15, 0.42)	0.542
CAR, Median (Q1, Q3)	1.94 (0.75, 4.08)	2.02 (0.76, 4.12)	1.91 (0.75, 3.71)	0.756
CLR, Median (Q1, Q3)	84.68 (30.04, 189.5)	87.5 (32.93, 191.09)	77.86 (27.9, 177.15)	0.641
CALLY, Median(Q1, Q3)	0.37 (0.16, 1.08)	0.35 (0.16, 1.04)	0.41 (0.16, 1.21)	0.634

HF, heart failure; CHD, Coronary atherosclerotic heart disease; CAU, urinary catheter; PICC, peripherally inserted central venous catheter; MV, mechanical ventilation; MDR, multidrug-resistant organisms; CRP, C-reactive protein; WBC, white blood cell count; LYM, lymphocyte count; MON, monocyte count; NEC, neutrophil count; HGB, haemoglobin; PLT, platelet count; GLU, glucose; CR, creatinine; TP, total protein; ALB, albumin; ALT, alanine aminotransferase; SII, Systemic Immune Inflammation Index; NLR, Neutrophil–Lymphocyte Ratio; PLR, Platelet–lymphocyte ratio; LMR, lymphocyte-monocyte ratio; PAR, Platelet–albumin ratio; NPAR, Neutrophil–albumin ratio; CAR, C-reactive protein–albumin ratio; CLR, C-reactive protein–lymphocyte ratio; CALLY, C-reactive protein-albumin-lymphocyte index.

### 3.2 Results of Cox regression analysis of the two groups in the training set

Univariate analyses were conducted on all variables within the training cohort dataset according to different outcomes. Results from univariate Cox regression analyses for variables across both groups indicated that the following factors constitute prognostic risk factors for ischaemic stroke patients following bloodstream infection: advanced age, concomitant pneumonia, HF, CHD, invasive procedures such as MV, reduced HGB levels, decreased ALB levels, and elevated CLR. Specific details are presented in **Table 2**.

**Table 2:** Results of univariate Cox regression analysis.

Characteristic	Total (n = 237)	Survival group (n = 203)	Death group (n = 34)	HR	95% CI	*P*-value
Male, N (%)	144 (61)	123 (61)	21 (62)	1.07	0.54, 2.14	0.841
Age, Median (Q1, Q3)	76 (67, 85)	76 (67, 84.5)	79 (72.25, 88.75)	1.03	1.00, 1.07	0.045
Combined underlying conditions						
Hypertensive, N (%)	188 (79)	160 (79)	28 (82)	1.26	0.52, 3.05	0.606
Pneumonia, N (%)	126 (53)	100 (49)	26 (76)	3.12	1.41, 6.89	0.005
HF,N(%)	42 (18)	26 (13)	16 (47)	4.82	2.46, 9.46	<0.001
CHD,N(%)	62 (26)	46 (23)	16 (47)	2.77	1.41, 5.44	0.003
Invasive procedure						
CAU,N(%)	136 (57)	108 (53)	28 (82)	3.77	1.56, 9.12	0.003
PICC,N(%)	137 (58)	110 (54)	27 (79)	3.03	1.32, 6.96	0.009
MV,N(%)	39 (16)	25 (12)	14 (41)	4.10	2.07, 8.12	<0.001
Mix infection, N (%)	18 (8)	15 (7)	3 (9)	1.18	0.36, 3.85	0.789
MDR,N(%)	125 (53)	104 (51)	21 (62)	1.80	0.85, 3.83	0.126
CRP, Median (Q1, Q3)	62.00 (27.64, 126.57)	58.57 (24.51, 124.54)	92.60 (55.67, 149.93)	1.00	1.00, 1.01	0.075
WBC, Median (Q1, Q3)	10.98 (7.12, 15.31)	10.72 (7.06, 15.04)	12.26 (8.25, 16.17)	1.02	0.98, 1.07	0.325
LYM, Median (Q1, Q3)	0.71 (0.44, 1.13)	0.78 (0.46, 1.14)	0.55 (0.25, 0.85)	0.63	0.34, 1.18	0.147
MON, Median (Q1, Q3)	0.43 (0.17, 0.73)	0.46 (0.18, 0.73)	0.24 (0.11, 0.85)	0.80	0.34, 1.88	0.604
NEC, Median (Q1, Q3)	9.04 (5.57, 13.65)	8.82 (5.48, 13.61)	11.12 (7.29, 14.26)	1.03	0.98, 1.07	0.216
HGB, Median (Q1, Q3)	100(79, 120)	102(81.5, 120.5)	80.5 (64.25, 110)	0.98	0.96, 0.99	<0.001
PLT, Median (Q1, Q3)	224 (156, 296)	226 (164, 307)	208.5 (130.5, 253.25)	1.00	0.99, 1.00	0.013
GLU, Median (Q1, Q3)	7.42 (6.05, 10.69)	7.32 (6.02, 10.07)	8.49 (6.83, 11.69)	1.01	0.96, 1.07	0.668
C R, Median (Q1, Q3)	79.7 (57.0, 119.0)	76.2 (55.6, 109.0)	113.5 (77.2, 192.5)	1.00	1.00, 1.00	0.011
TP, (Mean ± SD)	65.04 ± 8.43	65.53 ± 8.01	62.14 ± 10.27	0.95	0.91, 0.99	0.016
ALB, (Mean ± SD)	32.7 ± 5.63	33.5 ± 5.28	27.95 ± 5.35	0.85	0.80, 0.90	<0.001
ALT, Median (Q1, Q3)	21.1 (11.5, 38)	19.2 (11.6, 36.9)	22.4 (10.68, 49.27)	1.00	1.00, 1.00	0.038
SII, Median (Q1, Q3)	2714.38 (1225.95, 5417.38)	2667 (1187.96, 5193.10)	3227.34 (1457.52, 5730.93)	1.00	1.00, 1.00	0.226
NLR, Median (Q1, Q3)	12.35 (6.49, 24.27)	12.05 (6.35, 21.71)	21.96 (9.08, 37.19)	1.02	1.00, 1.03	0.007
PLR, Median (Q1, Q3)	298.46 (178.12, 495.74)	298.46 (179.63, 484.89)	294.53 (167.86, 533.12)	1.00	1.00, 1.00	0.120
LMR, Median (Q1, Q3)	1.87 (1.08, 3.60)	1.85 (1.07, 3.48)	2.13 (1.10, 3.85)	0.97	0.91, 1.03	0.277
PAR, Median (Q1, Q3)	6.51 (5.06, 9.63)	6.49 (4.99, 9.63)	6.73 (5.73, 8.90)	0.97	0.88, 1.07	0.556
NPAR, Median (Q1, Q3)	0.28 (0.17, 0.44)	0.26 (0.16, 0.43)	0.4 (0.21, 0.52)	4.27	1.51, 12.12	0.006
CAR, Median (Q1, Q3)	2.02 (0.76, 4.12)	1.88 (0.71, 3.84)	3.39 (1.80, 5.76)	1.24	1.10, 1.40	<0.001
CLR, Median (Q1, Q3)	87.50 (32.93, 191.09)	77.23 (29.61, 174.29)	171.29 (84.64, 372.12)	1.00	1.00, 1.00	<0.001
CALLY, Median (Q1, Q3)	0.35 (0.16, 1.04)	0.42 (0.20, 1.10)	0.15 (0.08, 0.36)	0.84	0.64, 1.10	0.207

CI, Confidence Interval; HR, Hazard Ratio; HF, heart failure; CHD, Coronary atherosclerotic heart disease; CAU, urinary catheter; PICC, peripherally inserted central venous catheter; MV, mechanical ventilation; MDR, multidrug-resistant organisms; CRP, C-reactive protein; WBC, white blood cell count; LYM, lymphocyte count; MON, monocyte count; NEC, neutrophil count; HGB, haemoglobin; PLT, platelet count; GLU, glucose; CR, creatinine; TP, total protein; ALB, albumin; ALT, alanine aminotransferase; SII, Systemic Immune Inflammation Index; NLR, Neutrophil–Lymphocyte Ratio; PLR, Platelet–lymphocyte ratio; LMR, lymphocyte-monocyte ratio; PAR, Platelet–albumin ratio; NPAR, Neutrophil–albumin ratio; CAR, C-reactive protein–albumin ratio; CLR, C-reactive protein–lymphocyte ratio; CALLY, C-reactive protein-albumin-lymphocyte index.

### 3.3 Results of the multivariate Cox regression analysis

Incorporating variables demonstrating statistical significance from the univariate Cox regression analysis into the multivariate Cox regression analysis revealed that reduced PLT and decreased serum ALB levels constitute independent risk factors influencing the prognosis of ischaemic stroke patients following bloodstream infection. Further details of the results are presented in **Table 3**.

**Table 3:** Results of the multivariate COX regression analysis.

Characteristic	HR ^a^	95% CI	*P*-value
Age	1.01	0.97,1.05	0.724
Pneumonia	0.42	0.16,1.08	0.070
HF	0.53	0.23,1.23	0.139
CHD	0.53	0.24,1.18	0.122
CAU	0.49	0.16,1.45	0.210
PICC	1.20	0.41,3.49	0.736
MV	0.78	0.32,1.87	0.574
HGB	1.00	0.98,1.02	0.797
PLT	1.00	0.99,1.00	0.049
CR	1.00	1.00,1.01	0.201
TP	0.98	0.93,1.04	0.525
ALB	0.89	0.80,0.99	0.024
ALT	1.00	1.00,1.00	0.090
NLR	1.02	0.99,1.05	0.294
NPAR	1.14	0.10,12.72	0.916
CAR	0.95	0.76,1.20	0.664
CLR	1.00	1.00,1.00	0.984

CI, Confidence Interval; HR, Hazard Ratio; HF, heart failure; CHD, Coronary atherosclerotic heart disease; CAU, urinary catheter; PICC, peripherally inserted central venous catheter; MV, mechanical ventilation; HGB, haemoglobin; PLT, platelet count; CR, creatinine; TP, total protein; ALB, albumin; ALT, alanine aminotransferase; NLR, Neutrophil–Lymphocyte Ratio; NPAR, Neutrophil–albumin ratio; CAR, C-reactive protein–albumin ratio; CLR, C-reactive protein–lymphocyte ratio.

Combine the four variables with *P* < 0.10 from the multivariate Cox regression analysis—pneumonia, PLT,ALB, and ALT into a multivariate non-penalised model, designated as Model 1. Using the 28-day prognosis outcome as the independent variable, the variance inflation factors of each variable in Model 1 were assessed to evaluate multicollinearity. The results showed that the variance inflation factors for the four variables in Model 1 were as follows: PLT: 1.045,ALB: 1.073,ALT: 1.006, Pneumonia: 1.057. The results of Model 1 in the training set multivariate Cox regression are presented in **Table 4**.

**Table 4:** Multivariate Cox regression analysis results for Model 1 in the training cohort.

Characteristic	HR	95% CI	*P*-value
pneumonia	3.00	1.34, 6.71	0.008
PLT	1.00	0.99, 1.00	0.029
ALB	0.86	0.80, 0.91	<0.001
ALT	1.00	1.00, 1.00	0.005

CI, Confidence Interval; HR, Hazard Ratio; PLT, platelet count; ALB, albumin; ALT, alanine aminotransferase.

### 3.4 LASSO regression for variable selection

**Figure 2:**
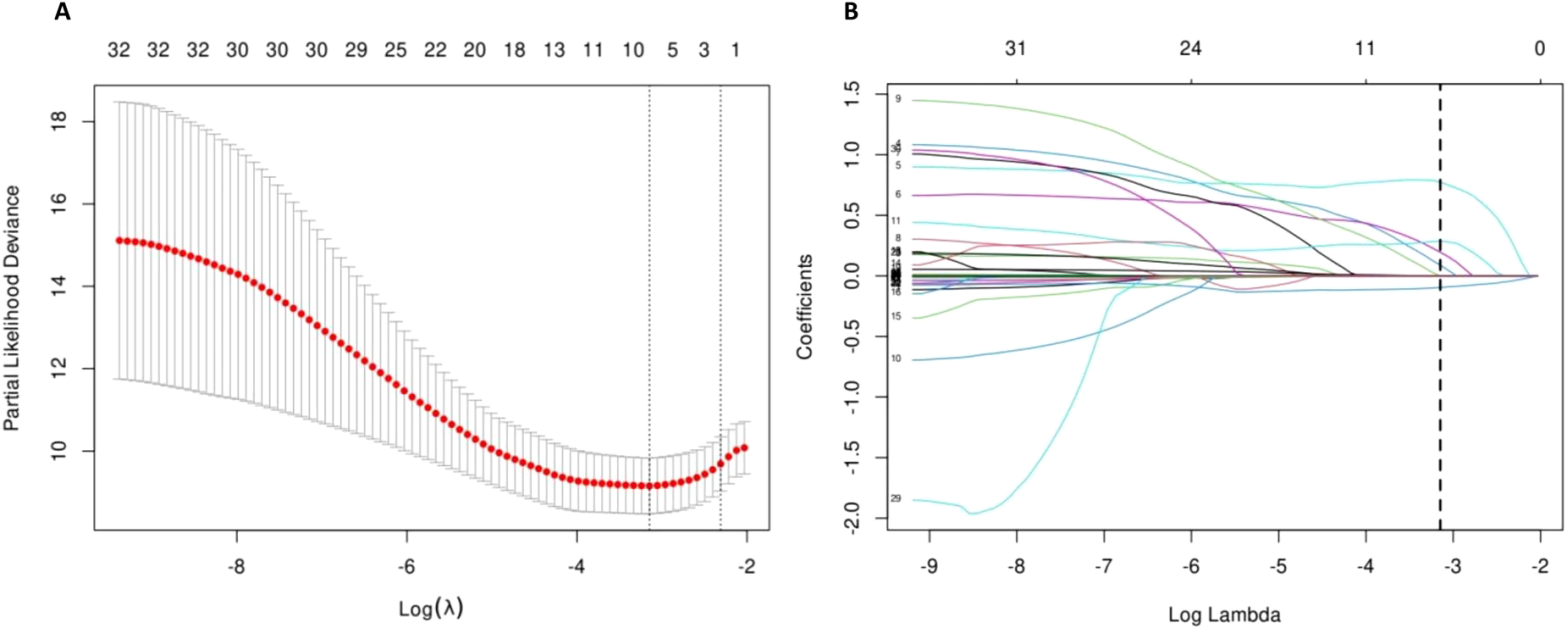
LASSO regression. **(A)** LASSO regression cross-validation plot; **(B)** LASSO regression coefficient path plot.

Given the limited dataset size and to mitigate issues of covariance and overfitting, we employed LASSO regression to validate variable selection for the multi-factor Cox regression analysis model. For all variables, LASSO regression analysis was conducted using 10-fold cross-validation. The LASSO regression coefficient paths and cross-validation results are illustrated in **Figures 1**, **2**. Model 2 was constructed using the variables corresponding to the lambda value that minimised the cross-validation mean squared error.

Model 2 comprises seven variables, including: whether pneumonia, HF, or CHD were present; whether mechanical ventilation was used; and the levels of ALB, ALT, and CLR indicators. Using the 28-day prognosis outcome as the independent variable, the variance inflation factors of each variable in Model 2 were assessed to evaluate multicollinearity. The results showed that the VIFs for the seven variables in Model 2 were: HF: 1.121, CDH: 1.090, MV: 1.181, ALB: 1.189, ALT: 1.038, CLR: 1.117. These seven variables were analysed using a multivariate Cox regression, with the results presented in **Table 5**.

**Table 5:** Multivariate Cox regression analysis results for Model 2 in the training cohort.

Characteristic	HR	95% CI	*P*-value
pneumonia	1.94	0.83, 4.50	0.124
HF	2.90	1.35, 6.23	0.006
CHD	1.78	0.83, 3.82	0.140
MV	1.34	0.62, 2.93	0.457
ALT	1.00	1.00, 1.00	0.031
ALB	0.85	0.79, 0.92	<0.001
CLR	1.00	1.00, 1.00	0.679

CI, Confidence Interval; HR, Hazard Ratio; HF, heart failure; CHD, Coronary atherosclerotic heart disease; MV, mechanical ventilation; ALB, albumin; ALT, alanine aminotransferase; CLR, C-reactive protein–lymphocyte ratio.

### 3.5 Comparison of the performance of the two models

Within the original training set, Model 2 achieved an AUC value of 0.861 and a C-index of 0.842 for the 28-day prognosis, surpassing Model 1’s AUC of 0.796 and C-index of 0.789. This demonstrates that Model 2 exhibits superior predictive accuracy to Model 1. Model 2 exhibited a Brill score of 0.089, lower than Model 1’s 0.094, indicating superior calibration performance. When comparing Model 1 as the older model against Model 2 as the newer model, the proportion of correctly reclassified cases in the newer model increased by 27.0% (Net Reclassification Improvement, NRI = 0.270).Following both the original training set and repeated five-fold cross-validation (performed three times), Model 2 consistently demonstrated a higher coefficient of determination than Model 1, indicating superior model fit. Both the RMSE and MAE of Model 2 were lower than those of Model 1 in the original training set and after three rounds of 5-fold cross-validation. This indicates that the average deviation between Model 2’s predictions and the actual observed values is smaller than that of Model 1. The specific numerical values for both models are presented in **Table 6**. Based on the above considerations, Model 2 was selected as the final model.

**Table 6:** Comparison of the performance of the two models.

Model	AUC	C-index	Brier score	NRI	Original training set			Cross Validation set		
					R-squared	RMSE	MAE	R-squared	RMSE	MAE
Model1	0.796	0.789	0.094	0.270	0.171	0.319	0.224	0.149	0.327	0.231
Model2	0.861	0.842	0.089		0.256	0.302	0.207	0.189	0.324	0.220

AUC, Area Under the Curve; NRI: Net Reclassification Improvement Index; RMSE, Root Mean Square Error; MAE, Mean Absolute Error.

### 3.6 Construction of the model nomogram

**Figure 3:**
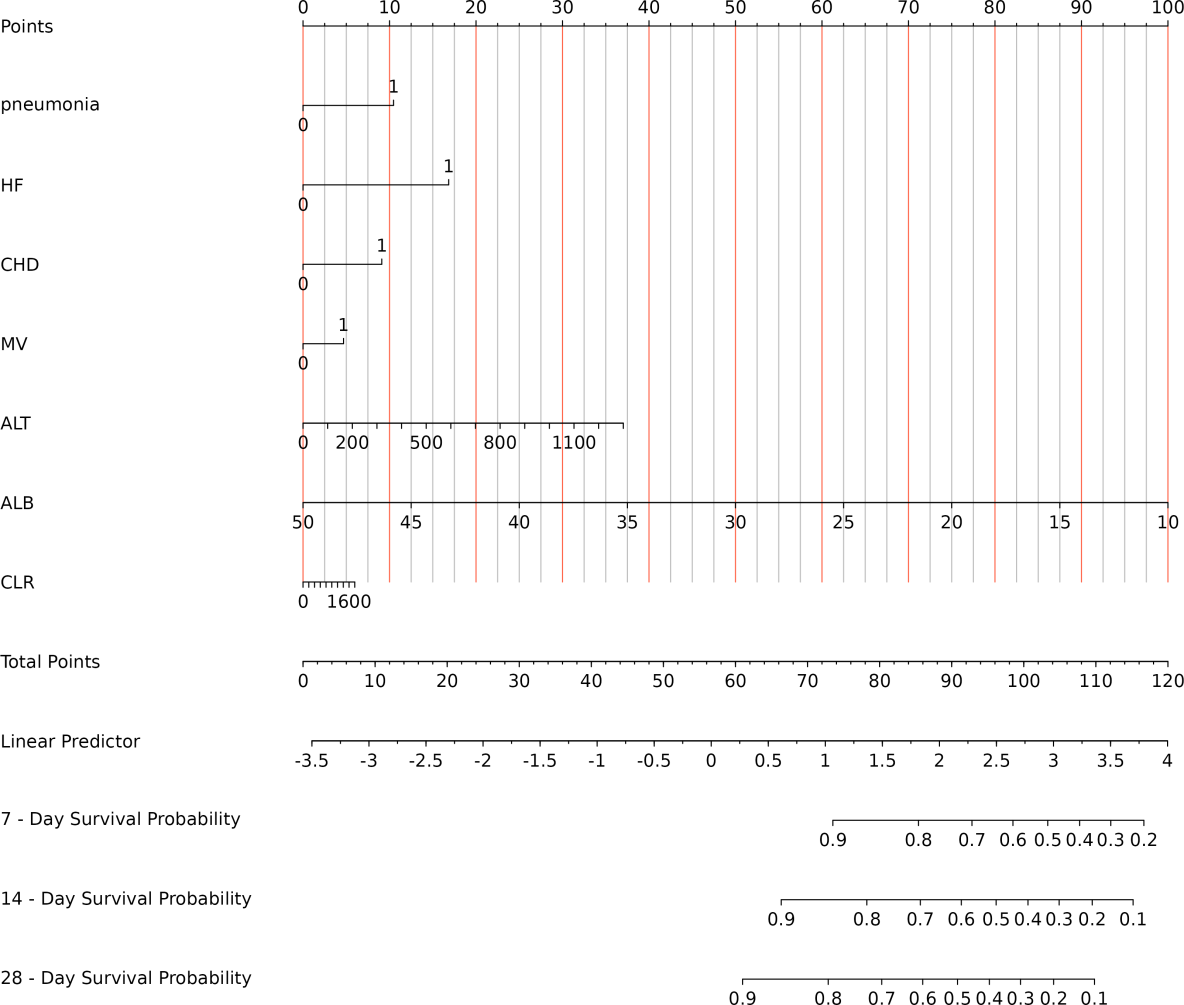
Nomogram prediction model. HF, heart failure; CHD, Coronary atherosclerotic heart disease; MV, mechanical ventilation; ALB, albumin; ALT, alanine aminotransferase; CLR, C-reactive protein–lymphocyte ratio.

The final model constructed using R software established a predictive framework for post-haemorrhagic stroke patients with bloodstream infections, generating a prediction column chart. The model indicates that total scores increase with the presence of concomitant pneumonia, HF, or CDH, MV use; elevated ALT and CLR values; and reduced ALB levels. Patients exhibiting higher total scores demonstrate a declining trend in survival rates (**Figure 3**). Suppose a patient presents with pneumonia and heart failure at the time of infection, without coronary heart disease or mechanical ventilation. Their C-reactive protein is 157.37 mg/L, lymphocyte count is 0.26 × 10^9^/L, albumin is 24.76 g/L, and alanine aminotransferase is 175.5 U/L. CLR: 605.269. According to the nomogram, the total score was 98 points, with a 7-day survival probability of 60%, a 14-day survival probability of 45%, and a 28-day survival probability of 35%. In actuality, this patient died 8 days after infection.

### 3.7 The AUC are compared at different time points to measure the accuracy the model

**Figure 4:**
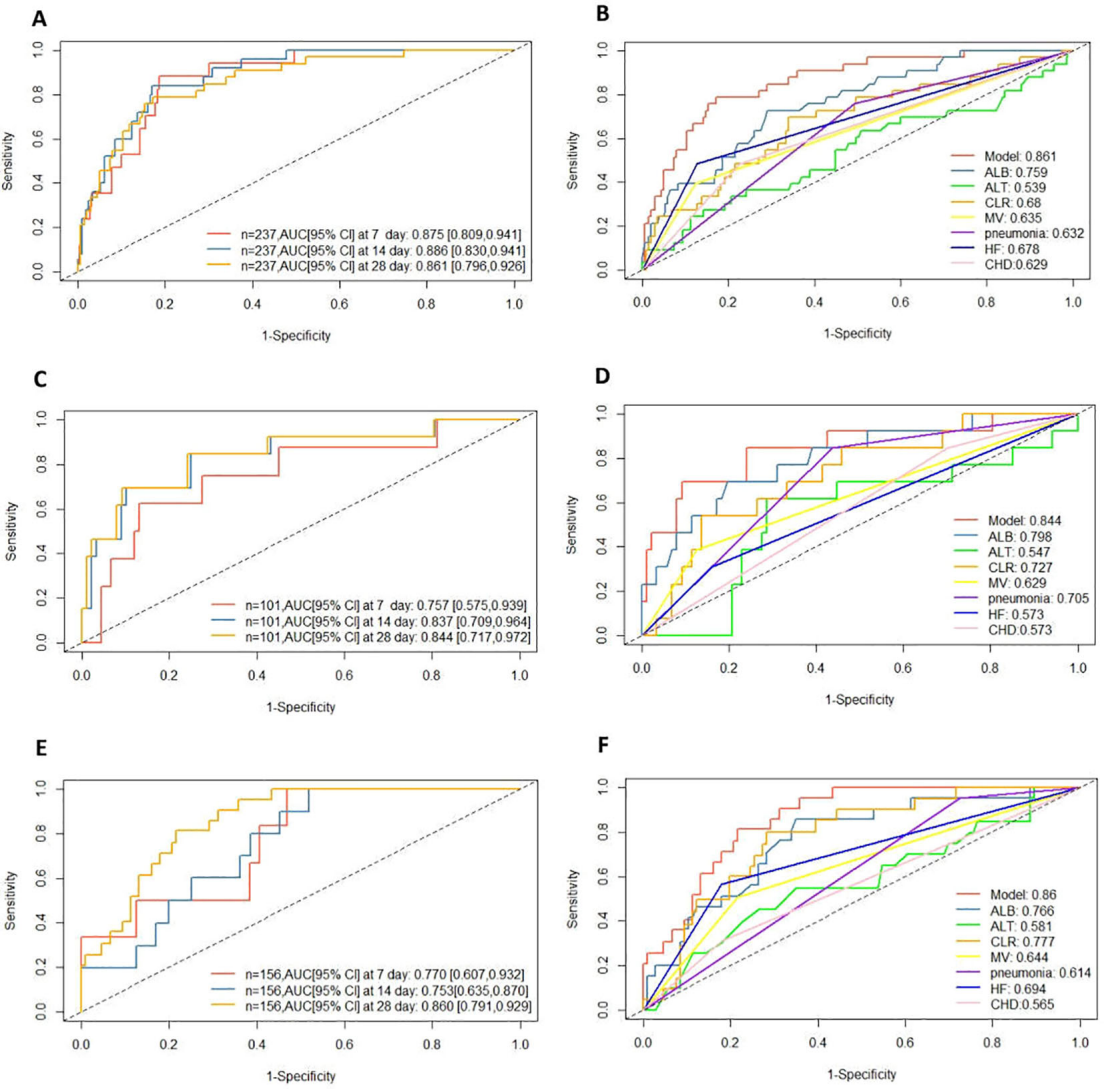
**(A)** ROC curves for different survival times in training cohort; **(B)** ROC curves at 28 days for different variables in training cohort; **(C)** ROC curves for different survival times in test colony; **(D)** ROC curves at 28 days for different variables in test cohort. **(E)** ROC curves for different survival times in external validation colony; **(F)** ROC curves at 28 days for different variables in external validation cohort. AUC: area under the curve; HF, heart failure; CHD, Coronary atherosclerotic heart disease; MV, mechanical ventilation; ALB, albumin; ALT, alanine aminotransferase; CLR, C-reactive protein–lymphocyte ratio.

The model was employed to predict survival rates at three time points—7 days, 14 days, and 28 days—for both the training and test datasets, yielding AUC values. Results demonstrated high discriminatory capability for survival prediction across all three time points in both datasets. Specifically, the AUC for predicting 14-day survival in the training dataset reached 0.886 (**Figure 4A**); while the highest AUC value of 0.844 was observed for the 28-day survival prediction in the test set (**Figure 4B**). Among the variables influencing the model, serum protein levels emerged as the most significant determinant for the 28-day prognosis model, yielding AUC values of 0.759 and 0.798 in the training and test sets respectively (**Figures 4C, D**).

In the external validation cohort, the model achieved AUC values of 0.770, 0.753, and 0.860 at days 7, 14, and 28 respectively. These AUC values indicate the model possesses robust survival discrimination capability throughout the entire 28-day period (**Figures 4E, F**).

### 3.8 The calibration curves are compared at different time points to measure stability of the model

**Figure 5:**
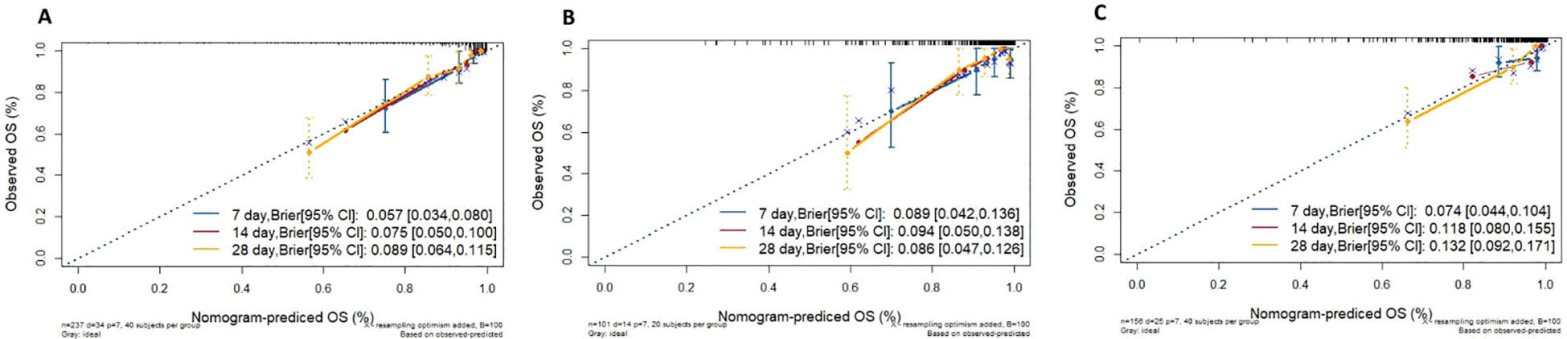
**(A)** Calibration curves for different survival time models in training colony. **(B)** Calibration curves for different survival time models in test colony. **(C)** Calibration curves for different survival time models in external validation colony. OS: overall survival.

Based on the number of samples in the training set, 40 samples were drawn each time with 100 resampling iterations. The results showed that the calibration curves for the 7 days, 14 days, and 28 days training sets, test sets, and external validation sets all formed a 45-degree angle. The model’s predicted probabilities closely matched the actual probabilities, indicating that the predictive model possesses excellent calibration capability (**Figure 5**).

### 3.9 DCA curves to assess the value of predictive modelling in clinical decision making

**Figure 6:**
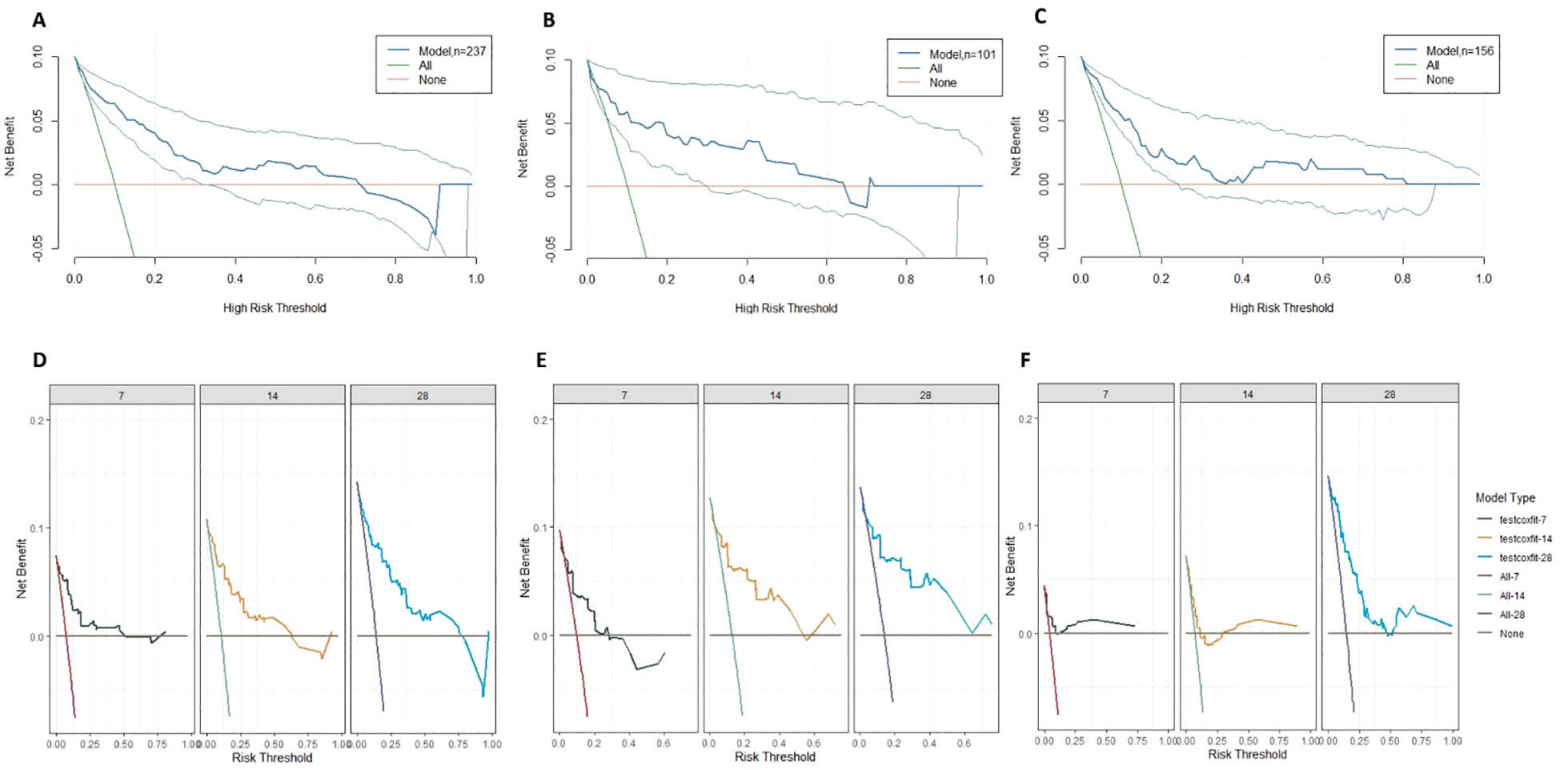
**(A)** 95% confidence interval for the DCA curve at 28 days prognosis in training colony; **(B)** 95% confidence interval for the DCA curve at 28 days prognosis in test colony; **(C)** 95% confidence interval for the DCA curve at 28 days prognosis in the external validation set; **(D)** DCA curves for different survival time models in training cohort; **(E)** DCA curves for different survival time models in test cohort; **(F)** DCA curves for different survival time models in external validation colony.

The clinical decision curves demonstrate that the model delivers favourable net benefit within the 5%-25% range at all three time points (7 days, 14 days, and 28 days) across all datasets. Notably, the 28-day decision curve exhibits a broader beneficial range, providing favourable net benefit across the 5%-60% spectrum (**Figure 6**). Interventions based on risk factors at 14 and 28 days yielded higher clinical benefits, indicating the model’s greater efficacy in identifying patients with poor prognosis during the later stages of infection. In the later stages, particularly within the 10%-25% risk range, this model identified a higher number of high-risk patients than the universal treatment strategy.

## 4 Discussion

This study focuses on the challenges in managing the prognosis of patients with bloodstream infections secondary to ischaemic stroke. A retrospective analysis was conducted on 494 eligible patients admitted between January 2022 and December 2024 across four medical institutions, including Guangdong Provincial Second Hospital of Traditional Chinese Medicine and Guangzhou Xinhai Hospital. In this cohort of 494 patients, 73 cases resulted in death, yielding a 28-day mortality rate of 14.8%. This rate substantially higher than the reported 28-day mortality of 3–6.4% observed in the general population of ischaemic stroke patients (Chen et al., 2020; Tu et al., 2023; Fan et al., 2025). As a common and severe complication of ischaemic stroke, bloodstream infection has been demonstrated to markedly exacerbate patients’ short-term mortality risk. Research indicates that following bloodstream infection, pathogens and their toxins disseminate via the circulatory system, triggering systemic inflammatory response syndrome. Concurrently, the massive release of inflammatory mediators such as Tumor Necrosis Factor-α(TNF-α) and Interleukin-6(IL-6) compromises the blood-brain barrier, exacerbating cerebral oedema. Circulatory failure induced by septic shock reduces cerebral perfusion, establishing a vicious cycle of ‘ischaemia-infection-re-ischaemia’. The pre-existing cerebral ischaemia and hypoxia in ischaemic stroke patients ultimately substantially elevate mortality risk (Joaquim et al., 2024; Bourhy et al., 2022).

The present study employed the LASSO regression algorithm, which automatically selects key variables through a regularisation penalty mechanism (Hepp et al., 2016). This approach effectively mitigates multicollinearity effects while eliminating irrelevant variables (such as gender and mixed infections). The study employed a triple validation system comprising ‘training set construction+validation set verification+cross-validation’ to comprehensively evaluate model performance. This established a complete logical chain of ‘construction-validation-application’, effectively demonstrating that prognostic models must balance statistical efficacy with clinical utility. It also provides a methodological framework for similar research. This study utilised the LASSO model to construct a nomogram, transforming complex regression calculations into an intuitive scoring system. Clinicians need only input whether the patient has concomitant pneumonia, HF, CHD, and whether mechanical ventilation is used, alongside measured values for ALB, ALT, and CLR. This facilitates the expeditious retrieval of the patient’s 7-day, 14-day, and 28-day survival probabilities, thereby substantially reducing the threshold for model utilisation. It provides a quantitative tool for prognosis assessment and risk management in patients suffering from ischaemic stroke and bloodstream infection.

In the constructed predictive model, patients with concomitant pneumonia, HF, and CHD exhibited significantly elevated overall prognosis scores and markedly reduced survival rates. Pneumonia is the most prevalent infectious complication following stroke (Westendorp et al., 2011). Pathologically, patients with ischaemic stroke already exhibit neurological impairment and immunosuppression. Concurrent pneumonia exacerbates systemic inflammatory responses, induces respiratory dysfunction, and intensifies cerebral hypoxia and ischaemia, thereby worsening prognosis (Westendorp et al., 2011). This has been shown to result in a worsened prognosis. This aligns with findings by Bustamante et al (Bustamante et al., 2017), who identified respiratory infections as an independent predictor of poor outcomes in stroke patients. The adverse effects of HF and CHD are closely linked to circulatory dysfunction: cardiovascular comorbidities are highly prevalent in stroke patients and significantly impact prognosis. The presence of HF exacerbates haemodynamic instability induced by sepsis, complicating fluid management and impairing peripheral perfusion—critical for cerebral and systemic functional recovery. Similarly, CDH indicates underlying vascular pathology and diminished cardiac reserve, limiting patients’ capacity to tolerate the metabolic demands of systemic infection and acute neuropathological stress (Harjola et al., 2020; Okobi et al., 2024). For patients suffering from ischaemic stroke, enhanced respiratory care is essential, including timed repositioning and back percussion, nebuliser therapy to thin sputum, and reducing the risk of aspiration pneumonia. Once pneumonia is confirmed, early administration of appropriate antibiotics based on sputum culture results is vital to prevent infection spread. For patients with concomitant HF or CHD, strict fluid intake control is essential. Diuretics and vasodilators should be employed to maintain cardiac stability, thereby averting cerebral hypoperfusion due to diminished cardiac output.

In this study, patients receiving MV exhibited elevated overall prognostic scores and reduced survival rates. This outcome may be explained by two factors: Firstly, MV, as an invasive procedure, compromises the respiratory mucosal barrier, thereby increasing the risk of bloodstream infections in patients with ischaemic stroke (Zhang et al., 2025); Secondly, patients requiring MV often present with severe respiratory dysfunction, indicating more severe underlying disease and inherently poorer prognosis (Asehnoune et al., 2023). Consequently, the model’s identification of ‘MV as a risk factor’ reflects not only the procedure’s direct impact but also indirectly indicates the severity of the patient’s underlying condition. Clinicians must therefore weigh the necessity of MV against the risk of infection, informed by an assessment of the patient’s respiratory function. For patients requiring mechanical ventilation (MV), it is imperative to adhere strictly to the Guidelines for the Prevention and Control of Ventilator-Associated Infections. The following measures are crucial for this purpose: firstly, the utilisation of non-invasive ventilation methods, such as nasal high-flow oxygen therapy, as alternatives to invasive ventilation, is paramount; secondly, there is a necessity for the regular replacement of ventilator circuits and the enhancement of oral care; thirdly, weaning and extubation of patients should be undertaken at the earliest possible opportunity, based on respiratory recovery, with the objective of minimising MV duration.

The model demonstrates that laboratory indicators indicating decreased ALB, elevated ALT, and increased CLR suggest poor prognosis. ALB is widely regarded as a core indicator reflecting nutritional status and immune function; In the present study, a decline in ALB levels was observed to be significantly correlated with diminished survival rates. It is evident that ALB fulfils a pivotal role in nutrition, yet its function extends beyond this domain, as it also plays a pivotal role in the regulation of infection through various mechanisms. These mechanisms encompass the maintenance of vascular colloid osmotic pressure, the binding of toxins, and the modulation of immune cell activity. Decreased ALB diminishes the body’s resistance to infection, facilitating its spread, while simultaneously weakening tissue repair capabilities, thereby increasing mortality risk (Jin et al., 2022). This aligns with findings by Dziedzic et al (Dziedzic et al., 2004), who observed that relatively higher serum ALB levels were associated with a reduced risk of adverse outcomes.

Elevated ALT typically indicates hepatic dysfunction, and in the model, increased ALT correlated with poor prognosis. During ischaemic stroke, cerebral ischaemia induces systemic alterations in peripheral organs. As a key metabolic organ, the liver must not only clear invading pathogens and their toxins; concurrently, the systemic inflammatory response triggered by infection (e.g., cytokine storms) exacerbates hepatic injury. Oxidative stress and inflammatory mediators arising from cerebral ischaemia reach the liver via the bloodstream, directly damaging sinusoidal endothelial cells and hepatocytes. The onset of a bloodstream infection has been shown to further exacerbate hepatic dysfunction, thereby establishing a vicious cycle of ‘infection-induced liver injury’ that elevates ALT levels (Chapman et al., 2009; Basic Kes et al., 2008). Upon entering the bloodstream, bacteria or their toxins activate Kupffer cells in the liver via Toll-like receptor 4, resulting in the release of substantial pro-inflammatory factors that induce hepatocyte apoptosis and necrosis. Concurrently, the septic state induced by infection reduces hepatic blood flow perfusion. This, in combination with hypoxic-ischaemic injury, further reduces hepatocyte metabolic capacity (de Dios et al., 2023; Pan et al., 2024). Conversely, hepatic dysfunction impairs the body’s anti-infective capacity: as the largest immune organ, the liver’s Kupffer cells clear circulating bacteria and toxins, while hepatocyte-synthesised complement and coagulation factors participate in immune defence. Elevated ALT levels are indicative of impaired hepatocyte function, which in turn reduces bacterial clearance efficiency and exacerbates infection progression (Abdeldyem et al., 2017). Hepatic dysfunction has been demonstrated to impair toxin metabolism and nutrient synthesis (Inderhees and Schwaninger, 2024). In addition, it has been shown to lead to coagulation disorders, which can further increase the risk of complications such as haemorrhage and infection spread. Coagulation abnormalities resulting from hepatic dysfunction have been demonstrated to exacerbate cerebral oedema following a stroke. In addition, infection-induced hypotension has been shown to further compromise hepatic and cerebral tissue perfusion. This process can ultimately result in a ‘death spiral’ of multiple organ failure, thereby exacerbating the poor prognosis of patients with ischaemic stroke and bloodstream infection.

The CRP level serves as an indicator reflecting the balance between inflammatory response and immune status. Elevated CRP in this study signalled poor prognosis. This elevation likely stems from two mechanisms: Firstly, elevated CRP levels, and secondly, reduced lymphocyte counts. CRP functions not only as an inflammatory marker but also directly contributes to tissue injury. Activation of the complement system results in the generation of membrane attack complexes, which has been demonstrated to exacerbate post-stroke cerebral inflammation and disrupt the blood-brain barrier. Concurrently, the process under investigation has been demonstrated to promote the expression of vascular endothelial cell adhesion molecules, thereby increasing thrombotic risk (Sproston and Ashworth, 2018; Badimon et al., 2018). Conversely, sustained inflammatory stress has been demonstrated to induce increased lymphocyte apoptosis and immune exhaustion. Research has demonstrated a decline in B cells, CD4 T cells, CD8 T cells, and natural killer cells following stroke, with CD4 T cell exhaustion exhibiting a strong correlation with increased infection rates (Vogelgesang et al., 2008; McCulloch et al., 2017). Lymphocytes are pivotal immune cells in the clearance of infection foci, and lymphopenia plays a critical role in the onset and progression of post-stroke infections (Wang et al., 2020). The depletion of these cells has been demonstrated to reduce bacterial clearance efficiency and impede infection control. Furthermore, persistent infection has been shown to exacerbate inflammation and lymphocyte depletion, creating a vicious cycle. It has been hypothesised that an elevated C-reactive protein (CRP) level may be indicative of an underlying inflammatory response, which, in turn, can result in an immunological imbalance. This, in turn, can lead to a worsening of the infection, and consequently, an elevation in the CRP level. The present study hypothesises that the inverse relationship between elevated C-reactive protein (CRP) and lymphopenia leads to an exponential increase in cluster of differentiation (CD) 4+ T-lymphocytes (CLR), with the magnitude of elevation more accurately reflecting the degree of inflammation-immunity imbalance. In this study, the median C-reactive protein (CRP) level (92.60 mg/L) in the mortality cohort was 1.6 times that of the survival cohort (58.57 mg/L), while the median absolute lymphocyte count (0.55 × 10^9^/L) was 0.71 times that of the survival cohort (0.78 × 10^9^/L). The combined effect of these two factors resulted in a median CLR of 171.29 in the mortality group, which was 2.2 times that of the survival group (77.23) (P<0.001). This magnitude of change was substantially greater than that observed for either single indicator alone, explaining why CLR demonstrated superior prognostic value compared to CRP or lymphocyte count considered individually. This aligns with findings by Lai et al (Lai et al., 2025), who identified elevated CLR as an independent risk factor for infection-related mortality in elderly patients with Acinetobacter baumannii bloodstream infections.

Model-screened liver function-related indicators, nutritional indicators, and inflammatory markers provide targets for symptomatic intervention and risk management in infected patients: Optimising nutrition and inflammation management with ALB as the core: For patients with hypoalbuminaemia, timely albumin infusion should be administered alongside enteral or parenteral nutritional supplementation to improve nutritional status. Concurrently, regular monitoring of markers such as CLR and ALT is essential. For patients with elevated CLR, immunomodulatory agents may be appropriately administered alongside antimicrobial therapy to bolster immune function. Those with elevated ALT should avoid hepatotoxic medications and receive hepatoprotective treatment where necessary to prevent further deterioration of liver function. Conversely, in the domains of patient risk management and infection prognosis management, clinical practice can leverage the model to assist clinicians in making rational decisions regarding healthcare resource allocation, thereby avoiding both over-intervention and under-intervention. This can be achieved through the dynamic adjustment of management strategies in response to ongoing monitoring. For instance, model predictions could be directly linked to clinical management tiers. Patients with a low risk profile (total score < 60) The management of the ward is to be conducted in accordance with the following protocol: Vital signs and infection markers (e.g. blood counts, CRP) are to be monitored on a daily basis.-Hepatic and renal function is to be reassessed tri-daily. Patients with an intermediate risk (total score: 60–80) The patient is to be transferred to a transitional care unit, where there will be an increase in the frequency of monitoring and the presence of dedicated nursing staff to ensure the implementation of infection prevention protocols. Patients considered to be at high risk (total score ≥80) Admission to the intensive care unit (ICU) is required, with multi-organ function monitoring being performed. The establishment of a multidisciplinary team comprising Neurology, Infectious Diseases, and Critical Care Medicine is imperative for the development of personalised treatment plans. It is imperative that dynamic monitoring is implemented in order to adjust strategies based on outcomes. Core model indicators (ALT, ALB, CLR) should be incorporated into daily monitoring protocols, and intervention intensity should be modified according to metric fluctuations. For instance, if a patient with a history of infection demonstrates a ≥30% reduction in CLR and ALT returns to within normal parameters following treatment, signifying an effective intervention, the current therapeutic regimen may be sustained. Should there be a persistent rise in CLR or a further increase in ALT, this would suggest inadequate infection control or the emergence of organ dysfunction. In such cases, it would be appropriate to escalate antimicrobial therapy or intensify hepatoprotective treatment in a timely manner. Furthermore, the implementation of a 28-day follow-up protocol for high-risk patients is imperative. This should encompass outpatient reviews or telephone follow-ups at 14 and 28 days post-discharge, with the objective of monitoring for the reappearance of infection symptoms and intervening promptly to avert adverse outcomes.

However, it is important to acknowledge certain limitations of the present study. This single-centre retrospective investigation, with a sample size of only 338 cases, may be subject to selection bias. For instance, the patient characteristics at Guangdong Provincial Second Hospital of Traditional Chinese Medicine may differ from those in other regional hospitals, potentially restricting the model’s generalisability. Furthermore, the study excluded certain potentially significant factors, such as patients’ neurological deficit scores and antibiotic treatment duration. The absence of these variables may result in an incomplete interpretation of prognosis by the model, necessitating their inclusion in subsequent research. Finally, this study focused solely on 28-day outcomes without assessing long-term survival. Future researchers should conduct multicentre, prospective studies with expanded sample sizes to further validate the model’s generalisability. Additionally, extending follow-up periods would enhance the assessment of the model’s long-term predictive efficacy.

## 5 Conclusion

This study successfully established a prognostic prediction model for patients with bloodstream infections following ischaemic stroke using the LASSO regression algorithm. A total of seven core risk factors were identified: pneumonia, HF,CHD, MV, decreased ALB levels, elevated ALT, and increased CLR levels. The model demonstrated excellent predictive performance and clinical utility. From a clinical application perspective, risk control measures proposed based on model variables—enhancing respiratory care for pneumonia, standardising MV procedures, and monitoring/intervening on indicators such as ALB, can specifically reduce the risk of adverse outcomes. The constructed cut-off diagram provides clinicians with a convenient prognostic assessment tool. Notwithstanding the limitations including single-centre recruitment and modest sample size, this study provides crucial evidence for precision prognostic management in patients with ischaemic stroke and bloodstream infections, laying the groundwork for future multicentre investigations.

## Code availability

The statistical software “R- version 4.3.1” was used.

## Data Availability

The dataset will be available upon request. Requests to access these datasets should be directed to Taoyuan Huang, huangtaoyuan1122@163.com.
